# Fine-tuning carbapenem resistance by reducing porin permeability of bacteria activated in the selection process of conjugation

**DOI:** 10.1038/s41598-018-33568-8

**Published:** 2018-10-15

**Authors:** Hoi-Kuan Kong, Qing Pan, Wai-U. Lo, Xuan Liu, Carmen O. K. Law, Ting-fung Chan, Pak-Leung Ho, Terrence Chi-Kong Lau

**Affiliations:** 1 Department of Biomedical Sciences, City University of Hong Kong, Hong Kong Special Administrative Region, Kowloon Tong, People’s Republic of China; 2 Department of Microbiology and Carol Yu Centre for Infection, The University of Hong Kong, Hong Kong Special Administrative Region, Kowloon Tong, People’s Republic of China; 3 School of Life Sciences, The Chinese University of Hong Kong, Hong Kong Special Administrative Region, Kowloon Tong, People’s Republic of China

## Abstract

Antibiotic resistance is an emerging public health issue. Plasmids are one of the popular carriers to disseminate resistance genes among pathogens. However, the response of plasmid-carrying bacteria to antibiotic treatment and how these bacteria evolve to increase their resistance remain elusive. In this study, we conjugated plasmid pNDM-HK to *E. coli* J53 recipient cells and selected survivors using different concentrations of the broad spectrum antibiotic meropenem. After selection, transconjugants conferred varying minimum inhibitory concentrations with respect to carbapenems. We sequenced and compared the transcriptomes of transconjugants that exhibited distinct carbapenem susceptibilities, and found that the loss of outer membrane proteins led to antibiotic resistance. Moreover, we identified a novel mutation, G63S, in transcription factor OmpR which moderates the expression of outer membrane proteins. The loss of porins was due to incapability of phosphorylation, which is essential for porin transcription and carbapenem resistance. We also characterized other genes that are regulated by *ompR* in this mutant, which contributed to bacterial antibiotic resistance. Overall, our studies suggest antibiotic pressure after conjugation might be an alternative pathway to promote antimicrobial resistance.

## Introduction

Development of antibiotic resistance among *Enterobacteriaceae* has become a worldwide health issue^1^. In the past, extended-spectrum β-lactamases (ESBLs), which hydrolyze extended-spectrum cephalosporins, have emerged and carbapenems were regarded as a last resort antibiotic^2^. However, the development of carbapenemases that can inactivate the carbapenems substantially increases the burden of clinical treatment at hospitals. New Delhi metallo-β-lactamase 1 (NDM-1) is one of the dominant carbapenemases. The NDM-1 gene (*bla*_NDM-1_) was first identified from a strain of *Klebsiella pneumoniae* in India^3^, and has since been found in various Gram-negative pathogens^4^. Conjugative transfer of plasmids is one of the major pathways to disseminate *bla*_NDM-1_ among bacteria, regardless of their species and class. In recent years, numerous plasmids carrying the NDM-1 gene, including both narrow (IncFII) and broad host range (IncM, IncA/C, IncH and IncX3) plasmids^5,6^, have been reported to associate with other classes of antibiotic-resistance genes, constraining therapeutic options for the infections caused by bacteria carrying these plasmids^7,8^.

Apart from antibiotic degradation enzymes, reduced expression of porins is a complementary mechanism to confer carbapenem resistance in bacteria^9^. Porins are outer membrane proteins that act as a selective barrier to protect bacteria from harmful substances and function as channels for molecules to diffuse into the cytoplasm. In *Escherichia coli* (*E. coli)*, OmpC, OmpF and PhoE are porins that control the influx of metabolites, amino acids and antibiotics^10^. Down-regulating these genes would reduce the permeability of porin, resulting in mitigated antibiotic resistance. Notably, clinical isolates with carbapenem resistance are often associated with porin loss^11,12^.

In the last decade, the relationship between gene regulation and antibiotic resistance, as well as the environmental factors that initiate gene moderation to activate antibiotic resistance pathways, have been widely studied^13^. It was suggested that incubating bacteria with sublethal concentration of antibiotics altered gene expression for adaptation and survival^14^. Moreover, antibiotics have been shown to act as the selective driver to influence the physiology of bacteria, such as conjugation dynamics^15^. Nevertheless, the effect from the selective pressure of antibiotics in terms of bacterial adaptation and their responses leading to further development of antibiotic resistance, particularly in the presence of antibiotic resistance plasmids, remain limited.

Previously, we isolated the first NDM-1 positive *E. coli* strain in Hong Kong and sequenced the corresponding plasmid pNDM-HK^16^. We found that the 90 kb plasmid contains a 55 kb backbone and a 28.9 kb variable region. The backbone shared 97% homology with pEL60 originated from the plant pathogen *Erwinia amylovora*, while the variable region encodes resistant determinants to β-lactams (*bla*_NDM-1_, *bla*_TEM-1_, *bla*_DHA-1_), aminoglycosides (*aacC2, armA*), sulphonamides (*sul1*) and macrolides (*mel, mph2*). It is in high homology to pCTX-M3 plasmid which has been associated with the dissemination of CTX-M type β-lactam resistance with the difference of a unique region of *bla*_NDM-1_. In order to understand the effect of antibiotics on bacteria carrying pNDM-HK, we conjugated pNDM-HK to *E. coli* J53 from a clinical isolate and selected using different concentrations of meropenem. The minimum inhibitory concentrations (MICs) of transconjugants was drastically changed under different conditions. Transconjugants selected by higher dosage of meropenem exhibited stronger carbapenem resistance. Transcriptomic analysis of these isogenic transconjugants indicated the development of stronger resistance was attributed to a novel point mutation G63S on the porin transcription factor, *ompR*, leading to the loss of porin. Our findings highlighted the importance of antibiotic selective pressure to bacterial physiology and their manipulations on gene expression for further developing stronger antibiotic resistance strains.

## Materials and Methods

### Bacterial strains and growth conditions

The *E. coli* strains and plasmids used in this study are listed in Table S1. DH5α and BL21(DE3) were used for cloning and overexpression of proteins, respectively. *E. coli* K-12 strains BW25113, JW2203, JW0912 and JW3368 were provided by the National BioResource Project at NIG^17^. Bacteria were grown in LB broth at 37 °C under shaking at 250 rpm. Antibiotic concentrations in growth media were applied as below: meropenem 0.12 µg/ml, ampicillin 100 µg/ml, kanamycin 25 µg/ml or chloramphenicol 25 µg/ml.

### Conjugation

Conjugation was performed using *E. coli* J53Az^r^ as the recipient. Donor strain *E. coli* (HK-01) were grown to late-exponential phase in LB and cell number was adjusted to 1.5 × 10^8^ cells/ml. Donor and recipient cells were combined in LB broth without antibiotic in a 1:2 donor-to-recipient ratio at 37 °C for 16 h. Transconjugants were selected using LB agar plates containing sodium azide (100 μg/ml) and different concentrations of meropenem (0.12, 0.5, 1 and 2 µg/ml). The transfer frequencies were obtained as the number of transconjugants per donor cell. After selection, all the transconjugants were cultured under the same concentration of meropenem (0.12 µg/ml).

### Growth curve measurement

Transconjugants were cultured in 50 mL LB at 37 °C with continuous shaking at 250 rpm. The optical density at 600 nm (OD_600_) was measured by Spectrophotometer (UV mini-1240) until stationary phase at every hour. Growth curve was plotted using absorbance 600 nm as y-axis and time as x-axis. Absorbance at each time point was calculated using the mean of biological duplicates. The bacteria’s doubling time of bacteria was determined by the period of time needed to grow from exponential phase to stationary phase.

### Plasmid and strain construction

DNA purification, restriction endonuclease cleavage, ligation and transformation were carried out according to the manufacturer’s protocols. In particular, TIANprep Rapid Mini Plasmid Kit (DP105) was applied for plasmid extraction based on the reference procedure. Coding sequence of *ompR* was amplified from transconjugants NDM-T21a and NDM-TC, and cloned into pET28a to generate His-WT-OmpR and His-G63S-OmpR, respectively. Mutation D55A of OmpR was constructed using two-primer site-directed mutagenesis. Briefly, two complementary primers encoding 50 nucleotides with the desired mutation sites in the middle of primers were utilized. The PCR reaction involved 20 cycles at 98 °C (10 seconds), 42 °C (1 minute) and 72 °C (7 minutes) using iProof DNA-polymerase (Bio-Rad) with pTL39 (pET28a-ompR) as template. The reaction was then digested with *Dpn* I (NEB) to remove the template plasmid, purified and transformed into DH5α. For EnvZc expression, C terminal (R180-G450) of EnvZ was subcloned into pGEX-6P-1 to generate GST-EnvZc and transformed into BL21 (DE3). Transcription units of *ompR* in NDM-T21a and NDM-TC, ranging from −250 to +840 bp, were cloned into single-copy vector pNN387 to generate pTL74 and pTL75.

### RNA extraction and library construction

Transconjugants NDM-T21a and NDM-TC were grown to early stationary phase (OD_600_ ~2). Cell pellets were mixed with 3 volumes of TRIzol reagent (Invitrogen), and the RNA was extracted by adding 1 volume of chloroform followed by centrifugation. Total RNA was precipitated in isopropanol, and the concentration was measured using NanoDrop ND-1000 spectrophotometer (Thermo). The quality of RNA was determined by TAE agarose gel electrophoresis.

Messenger RNA was enriched and separated from small RNA using a mirVana™ miRNA Isolation Kit (Life Technologies) and further subjected to a MICROBExpress Kit (Ambion) and a Ribo-Zero rRNA Removal Kit (Epicentre) to remove ribosomal RNA according to the manufacturer’s instructions. The concentration of mRNA was measured by ND-1000 (Thermo) and the quality was determined by a Bioanalyzer (Agilent) using an RNA 6000 Pico Kit (Life Technologies).

### RNA sequencing and data analysis

The rRNA-depleted RNA was used to construct the library with the Ion Total RNA-Seq Kit v2 (Ambion) according to the manufacturer’s instructions. Libraries were next sequenced using the ion Torrent sequencing platform on Ion 318 Chips (Life Technologies). Reads were mapped to reference genome *E. coli* str. K-12 substr. MG1655 (GenBank accession NC_000913.3) using TMAP^18–22^. Those mapped sequencing reads were visualized using an Integrated Genome Viewer (IGV) 2.3.34^23^. The number of reads mapped to each gene was normalized to the gene expression level according to gene length, sum of read length, and the total number of mapped reads.

### Protein purification

Bacteria with the plasmids encoding the desired gene were grown to OD600~0.6 at 37 °C, and 1 mM IPTG was added to induce protein expression. After shaking for 4 hours, cells were harvested by centrifugation and re-suspended in a lysis buffer (20 mM Tris-HCl, 150 mM NaCl, 0.5% Triton X-100, 0.4 mM PMSF and 2 mM β-mercaptoethanol, pH 7.5). For the purification of OmpR, bacteria were lysed by sonication and protein was purified using a TALON Cobalt column (Clontech).

High salt wash buffer (20 mM Tris-HCl, 500 mM NaCl, 20 mM imidazole, 2 mM β-mercaptoethanol, pH 7.5) and low salt wash buffer (20 mM Tris-HCl, 150 mM NaCl, 20 mM imidazole and 2 mM β-mercaptoethanol, pH 7.5) were used to eliminate non-specific binding. The OmpR protein was eluted by elution buffer (20 mM Tris-HCl, 150 mM NaCl, 250 mM imidazole and 2 mM β-mercaptoethanol, pH 7.5).

For purification of EnvZ, supernatant was loaded on the glutathione column (GE healthcare), washed with buffer (20 mM Tris-HCl, 350 mM NaCl and 2 mM β-mercaptoethanol, pH 7.5) and eluted by elution buffer (20 mM Tris-HCl, 150 mM NaCl, 10 mM reduced glutathione and 2 mM β-mercaptoethanol, pH 7.5). All purified proteins were dialyzed against storage buffer (20 mM Tris-HCl, 200 mM NaCl and 10% glycerol, pH 7.5) at 4 °C overnight. Protein purity and concentration were determined by A280/A260 and SDS-PAGE (Purity > 95%).

### EnvZ phosphorylation assay

To prepare the ^32^P labelled phosphorylated form of EnvZ, EnvZ (1 µM) was phosphorylated in a 150 µL buffer containing 50 mM Tris (pH 7.5), 50 mM KCl, 5 mM CaCl_2_, 2 mM β-mercaptoethanol, and 35 µCi [γ-^32^P] ATP at room temperature for 20 minutes. The reaction mixture was then passed through a Centri-Spin 10 column (Princeton Separations) to remove excess [γ-^32^P] ATP. To perform the assay, OmpR or mutant (1 µM) was mixed with the phosphorylated EnvZ mixture. The reaction was kept on ice and terminated at the indicated time by adding stop buffer (124 mM Tris-HCl (pH 6.8), 20% glycerol, 4% SDS, 8% β-mercaptoethanol and 0.025% bromophenol blue). The products were separated using 12% SDS-PAGE, and the image was analyzed by phosphor-imager.

### Quantitative Real-time PCR (qRT-PCR)

To remove the contaminated DNA, RNA was treated with 2U TURBO DNase (Ambion) at 37 °C for 30 min twice, and incubated with 1/10 (v/v) inactivation reagent (Ambion) to inactivate DNase. For quality control, resulting RNA was subjected to a DNA contamination test by PCR using *gapA* primers. One µg qualified RNA was next reverse-transcribed into cDNA by Superscript III First-Strand Synthesis System (Life Technologies) according to the instructions of the manufacturer. Primers for qRT-PCR were designed with Primer3 software^24^ and listed in Table S2. The gene *gapA* served as the endogenous control for normalization. The reaction was set up with 5 µl Power SYBR Green PCR Master Mix (Life Technologies), 1 µl cDNA, 2 µM forward and reverse primers, and appropriate nuclease-free water to a total volume of 10 µl per reaction. The PCR was run on a 7500 Fast Real-time PCR System (ABI) with a program of 95 °C 5 min for 1 cycle, and 95 °C 15 s, 60 °C 1 min for 40 cycles. Each qRT-PCR analysis was performed in triplicate, whereas expression levels were calculated by Ct values using the formula 2^−△△Ct^.

### Western Blot

Around 2 × 10^7^ cells were used for sodium dodecyl sulfate−polyacrylamide gel electrophoresis (SDS−PAGE). Following that, proteins were transferred to the nitrocellulose membrane (Bio-Rad) with transfer buffer (25 mM Tris, 192 mM glycine and 10% methanol) at 120 V for 90 min. The membrane was then blocked with 5% nonfat milk in TBST solution (20 mM Tris, 150 mM NaCl and 0.1% Tween-20) at room temperature for 1 hour. After blocking, the membrane was immersed with polyclonal anti-NDM-1 antibody (Novus) and subsequently incubated with the goat anti-rabbit IgG HRP-conjugated secondary antibody (BioRad). Monoclonal mouse anti-GAPDH antibody (Cell Biolabs) was utilized to quantify the loading amount. Signals were obtained using a LAS 4000 chemiluminescent imager (Fuji) with the addition of Clarity™ western ECL substrate (Bio-Rad).

### Agar Disc Diffusion Assay

Peptide alafosfalin (L-alanyl-L-aminoethylphosphonic acid) was purchased from Sigma Aldrich. Alafosfalin was dissolved in nuclease-free water with the desired concentration and was loaded onto 6 mm filter paper discs (Whatman). Bacterial suspension was diluted from overnight culture to a density of 1.5 × 10^8^ cfu ml^−1^. Paper discs containing various concentrations of peptide were placed on LB agar inoculated with bacteria, where agar plates were kept at 37 °C for 16 hours. The diameters of inhibition zones were calculated from biological triplicates.

### Antimicrobial susceptibility test

Minimum inhibitory concentrations (MICs) of bacteria to doripenem, ertapenem, imipenem and meropenem were determined by Etest strips (AB Biodisk, Solna, Sweden). The susceptibility results were interpreted according to the Clinical and Laboratory Standards Institute (CLSI) guideline 2018 with *E. coli* strain ATCC 25922 as quality control of the susceptibility test.

## Results

### Dependence of minimum inhibitory concentration of transconjugants on antibiotic selection concentration

In order to interpret the effect of antibiotic on selection process, the plasmid was firstly conjugated from the clinical isolate (*E.coli*, HK-01) to *E.coli* J53 and selected with four different concentrations of meropenem. The transfer frequencies and MICs of the transconjugants were measured and compared in Table 1. After conjugation, MIC of transconjugant was slightly enhanced from 0.75 to 4 μg/ml under low dosage selection, which was possibly due to different host backgrounds. Interestingly, transconjugants selected from higher concentration of meropenem (1 and 2 µg/ml) possessed extremely strong antibiotic resistance to imipenem, meropenem, ertapenem, and doripenem. Notably, these two selection concentrations were higher than the MIC of the donor strain HK-01.

**Table 1:** MICs and conjugation frequencies of different pNDM-HK transconjugants, donor and recipient strain.

Strains	Antibiotic used for selection (concentration µg/ml)	Transfer frequency	MIC (µg/ml)			
			IPM	MEM	ETP	DOR
Donor, HK-01			0.75	0.75	1.5	1.5
Recipient J53			0.19	0.016	0.006	0.016
Transconjugants						
NDM-T21a	Meropenem (0.12)	1.8 × 10^−3^	8	4	6	4
NDM-T27b	Meropenem (0.5)	1.8 × 10^−4^	8	6	4	4
NDM-T4a	Meropenem (1)	2.7 × 10^−6^	>32	>32	>32	>32
NDM-TC	Meropenem (2)	2.1 × 10^−8^	>32	>32	>32	>32

Abbreviation:

IMP, imipenem; MEN, meropenem; ETP, ertapenem; DOR, doripenem.

### Transcriptomic analysis identified G63S OmpR mutant in NDM-TC

To determine whether NDM-1 contributed to the antibiotic resistance of transconjugants, we measured the protein level of NDM-1 in both NDM-T21a and NDM-TC strains which expressed the lowest and highest MICs respectively. As shown by Western blot analysis (Fig. S1), no significant difference was observed in both strains, indicating the increment of MIC in NDM-TC was not due to the antibiotic degrading enzyme (NDM-1) overexpressed from the plasmid. To unravel the carbapenem resistance mechanism of NDM-TC, we sequenced the transcriptomes (RNA-seq) of NDM-T21a and NDM-TC, and compared their expression profiles. A total number of 1,501,725, and 1,765,361 reads were obtained for NDM-T21a and NDM-TC, respectively. Raw reads with low abundance were filtered to reduce random errors. Sixty percent of total reads were mapped to the coding region and others were mapped to tmRNA and non-coding RNAs (Fig. S2a). We compared the expression levels between NDM-T21a and NDM-TC by log_2_(NDM-TC/NDM-T21a). Genes with fold changes larger than +2 or smaller than −2 are considered as differentially expressed. For chromosomal genes, 43 of them were repressed, whereas 77 genes showed up-regulation among NDM-TC, which account for 5% of the mapped genome (Fig. S2b). In addition, differential expressions were also found in plasmid-encoded genes among two transconjugants. A total of 5 genes of plasmids were significantly altered in NDM-TC as shown in Fig. S2c, suggesting a portion of metabolic pathways were evolved in NDM-TC during the selection process. Chromosomal and plasmid-encoded genes with differential expression were listed in Datasets S1 and S2 respectively.

**Figure 1:**
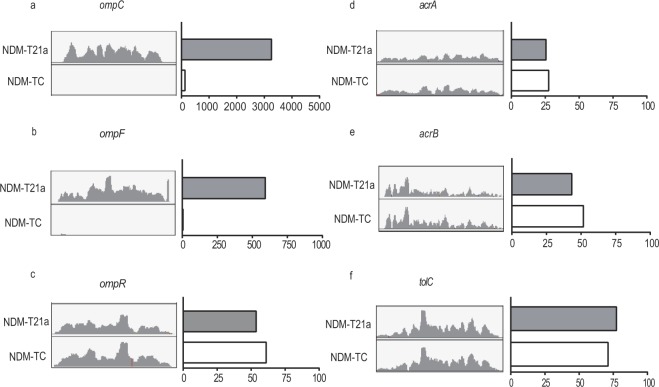
Read-count of genes related to the outer membrane protein and efflux pump in RNA-Seq. The read-count of (**a**) *ompC*, (**b**) *ompF*, (**c**) *ompR*, (**d**) *acrA*, (**e**) *acrB* and (**f**) *tolC* in transconjugants NDM-T21a (grey column) and NDM-TC (white column) are shown. IGV-captured diagrams with the same scale are placed next to the bar chart.

In our sequencing results, gene expression level of β-lactam resistance genes such as *bla*_TEM-1_ was similar in both NDM-T21a and NDM-TC, indicating that the enhancement of MIC in NDM-TC was not attributed to other β-lactamases (Fig. S3). Since drug susceptibility of bacteria can be controlled by the membrane proteins, we then compared the expression level of genes related to import and export pathways. Interestingly, a significant down-regulation of porin genes (*ompC* and *ompF*) up to 5-fold were observed in NDM-TC compared with NDM-T21a (Fig. 1). On the other hand, most efflux pump genes including *acrA*, *acrB* and *tolC* showed similar expression in all transconjugants. The expression levels of porin genes among two transconjugants were further quantified by qRT-PCR as shown in Fig. S4 while the fold change of efflux pump genes were listed in Dataset S3. Notably, reducing porin expression was reported as one of the major mechanisms of cephalosporin and carbapenem resistance^9,10^ and thereby, possibly resulted in high MIC of NDM-TC. To further investigate the regulatory mechanism of porins, we sequenced the whole transcriptional cassettes of *ompC* and *ompF*, including the leader and coding sequences. However, no genetic insertion, mutation or deletion was found in any region of these two genes. On the other hand, we identified a G183A nonsynonymous mutation in the coding region of *ompR*, resulting in an amino acid substitution from glycine to serine at position 63 (G63S) in OmpR protein (Fig. S5). OmpR is a transcription factor that regulates the expression of *ompC and ompF*.

### G63S OmpR abolishes *ompC* and *ompF* expression

**Figure 2:**
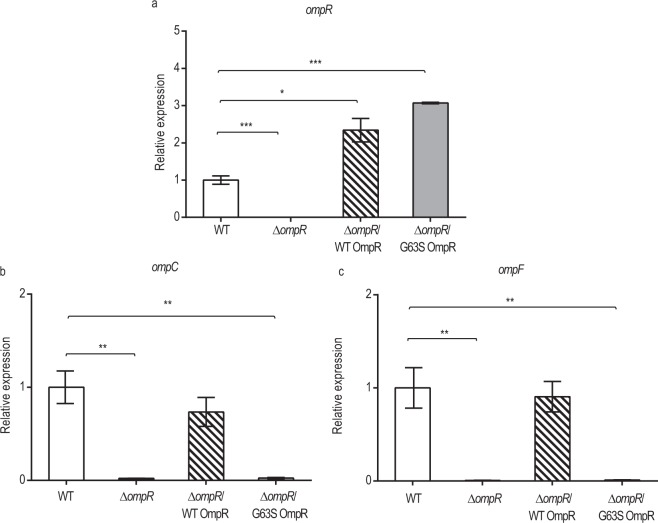
Transcription of (**a**) *ompR*, (**b**) *ompC* and (**c**) *ompF* in wild type and OmpR mutant obtained in qRT-PCT. Relative gene expressions are shown in wild type *E.coli* (white column), in the absence of *ompR* (black column), wild type OmpR recovered in null *ompR* allele (hatched column) and G63S OmpR recovered in null *ompR* allele (grey column). Vertical error bars show the standard deviations of biological triplicates. The expression level obtained in wild type was taken as a reference for comparison. ^*^P-value < 0.05; ^**^P-value < 0.01; ^***^P-value < 0.001.

To study the correlation between *ompR* G183A mutation and porin expression, we compared the expression of *ompC* and *ompF* in null *ompR* allele (∆*ompR*) and mutants recovered from either wild type (∆*ompR*/WT) or G63S OmpR (∆*ompR*/G63S). As shown in Fig. 2a, both wild type and G63S OmpR complements were successfully expressed in the ∆*ompR* strains. Moreover, overexpression of wild type OmpR completely restored the expression of *ompC* and *ompF*. On the other hand, no effect was observed in ∆*ompR* strains complemented with G63S OmpR (Fig. 2b,c). This finding demonstrated that G63S OmpR was not able to activate gene expression of porins.

### Failure of G63S OmpR phosphorylation by EnvZ

**Figure 3:**
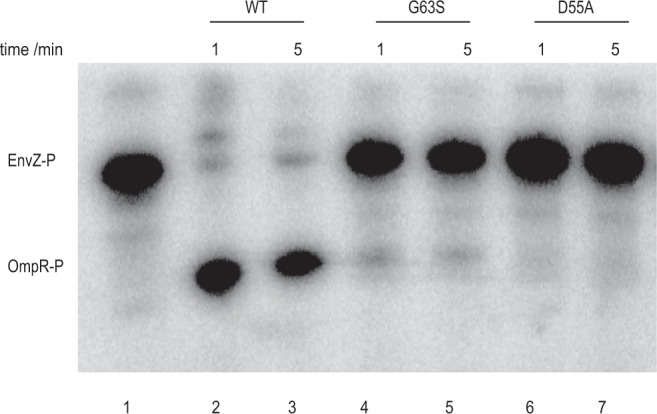
Phosphorylation of wild type, G63S and D55A OmpR by truncated EnvZ. Around 1 µg of EnvZ was autophosphorylated by ^32^P-ATP in lane 1. Approximately 0.28 µg of wild type OmpR (lanes 2–3), G63S mutant (lanes 4–5) or D55A mutant (lanes 6–7) were mixed with phosphorylated EnvZ. The reaction was terminated by adding stop buffer at 1 minute (lanes 2, 4 and 6), or 5 minutes (lanes 3, 5 and 7). The molecular weight ladder is marked on the right column. Full image is shown in Supplementary Figure S6.

Porin expression is mainly controlled by a signal transduction system in which sensor kinase EnvZ interplays with the response regulator OmpR. To activate porin transcription, the phosphorylated form of EnvZ is required to transfer the phosphoryl group to aspartic acid 55 (D55) of OmpR. The phosphorylation of OmpR induces the dimerization of the monomer, which then enhances its affinity to *ompC* and *ompF* transcription factor binding sites for gene activation. Since Glycine 63 of OmpR is located at the N-terminal phosphorylation domain, G63S mutation possibly affects the phosphorylation of OmpR. To find out, a recombinant and purified truncated form of EnvZ was used to test the phosphorylation of either wild type or G63S OmpR. The reaction process was monitored at 1 and 5 minutes. As shown in Fig. 3, the EnvZ was labelled with ^32^P-ATP by autophosphorylation (lane 1) and D55A OmpR mutant^25^ was used as a negative control (lanes 6 and 7). In lanes 2 and 3, the phosphorylated EnvZ (EnvZ-P) transferred the [^32^P] phosphoryl group to wild type OmpR after one minute of incubation, but no phosphorylation was observed in G63S OmpR (lanes 4 and 5). Phosphorylation of OmpR is essential to promote the binding on the promoter of *ompC* and *ompF*^25^, as well as to initiate gene transcription. G63S OmpR possibly fail to initiate porin transcription, resulting in carbapenem resistance in NDM-TC.

### Modeling of G63S OmpR

**Figure 4:**
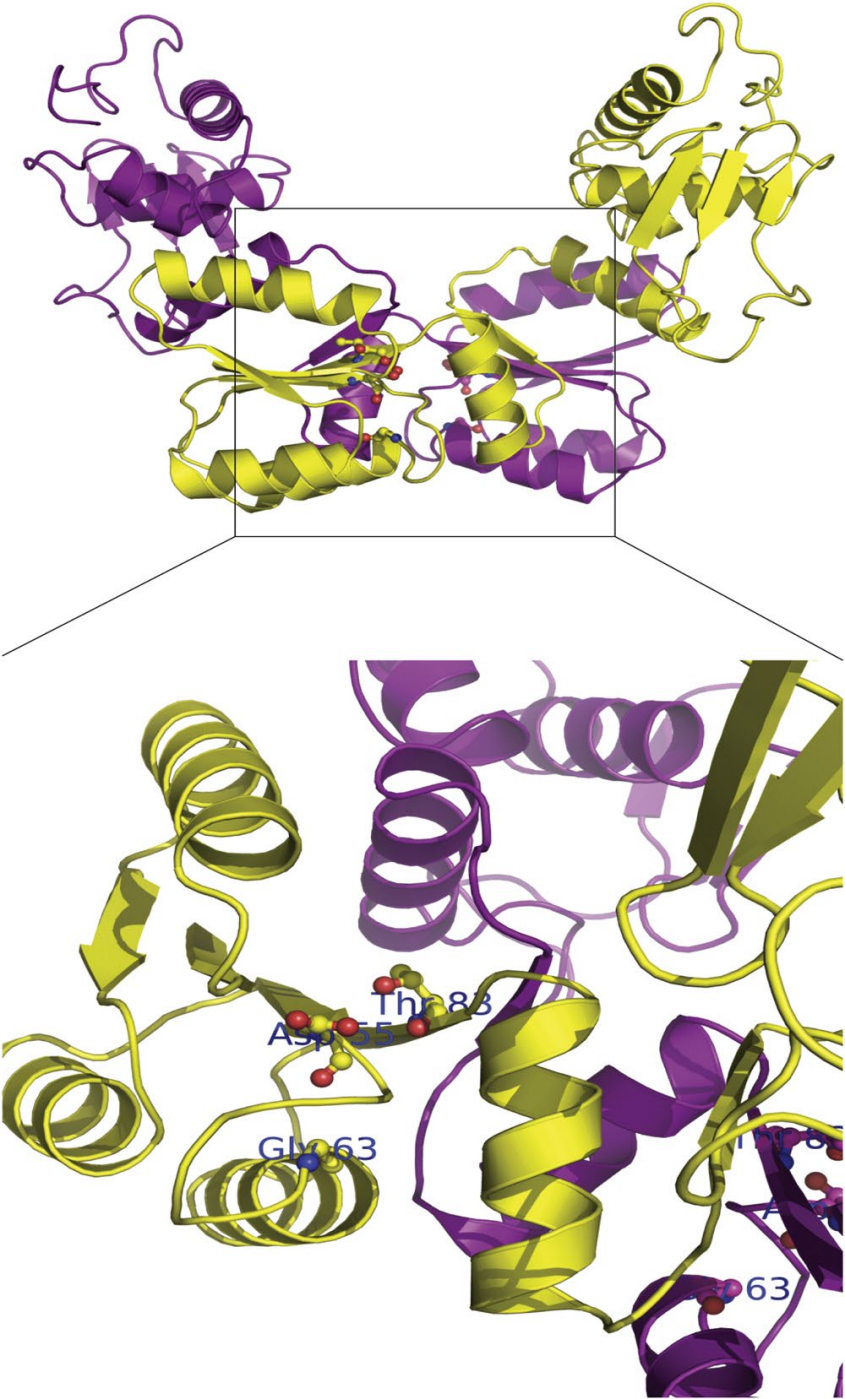
Molecular model of OmpR. The dimer form of OmpR structure and the zoom-in region of the phosphorylation site Asp55 are shown. The monomer of OmpR is shown in yellow and magenta color. The residues Asp55, Gly63 and Thr83 are highlighted in ball-and-stick format and colored by element (carbon, gray; nitrogen, blue; oxygen, red). The figures were produced using Discovery Studio visualizer (Accelrys).

To further understand the molecular basis of G63S mutation restricting OmpR phosphorylation, we built the model of G63S OmpR by homology modelling with the C- and N- terminal domains structures of OmpR in *E. coli* (1ODD and 1OPC) using SWISS-MODEL. As shown in Fig. 4, aspartic acid at position 55 (Asp55), the residue that undergoes phosphorylation, is in close proximity with Glycine 63 (G63) and Threonine 83 (T83). Mutation of T83 has been shown to inhibit the phosphorylation of Asp55. The change of glycine to serine could introduce a charge and steric hindrance effect at the phosphorylation site of OmpR, thereby inhibiting the reaction.

### Contribution of OmpR to carbapenem resistance

Loss of porin restricts the influx of drug to the periplasm, and thereby enhances antibiotic resistance^9^. Mutations in *ompC* and *ompF* have often been identified in drug-resistant pathogens^11,26^ while *ompR* mutations have been reported to promote carbapenem resistance in cephalosporinase producing bacteria^27–29^. However, the combinational effect of *ompR* mutation and the presence of carbapenemase has rarely been described. In order to study this effect, we transformed pNDM-HK into null *ompC*, *ompF* or *ompR* allele of *E.coli* K-12 strains and measured their MICs. As shown in Table S3, all strains carrying pNDM-HK exhibited at least a 100-fold increase of carbapenem resistance compared with their corresponding isogenic strains. Moreover, the effect of carbapenem resistance became stronger in null *ompR* and G63S *ompR* mutants, suggesting the synergistic effect between *ompR* and carbapenemase in antibiotic resistance.

### Regulation of other metabolic pathways by OmpR

**Figure 5:**
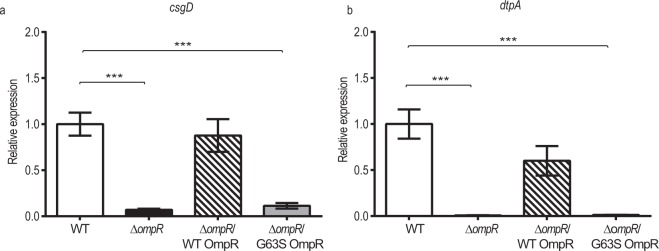
Transcription of (**a**) *csgD* and (**b**) *dtpA* in wild type and OmpR mutant obtained in qRT-PCR. Relative gene expressions are shown in wild type *E. coli* BW25113 (white column), in the absence of *ompR* (black column), wild type OmpR recovered in null *ompR* allele (hatched column), and G63S OmpR recovered in null *ompR* allele (grey column). Vertical error bars show the standard deviations of biological triplicates. The expression level obtained in wild type is set as reference for comparison. ^*^P-value < 0.05; ^**^P-value < 0.01; ^***^P-value < 0.001.

In addition to the alleviated expression of porin contributing to the carbapenem resistance in NDM-TC, down-regulation of other genes including curli subunit gene D (*csgD)* and tripeptide permease A (*dtpA)* were observed in the sequencing result, and their gene expression levels were further validated by qRT-PCR. Notably, expression of *csgD* and *dtpA* were controlled by OmpR regulon^30,31^. To determine whether G63S OmpR has any regulatory effect on these genes, we measured the expression level of these genes in ∆*ompR*/WT OmpR and ∆*ompR*/G63S OmpR strains. As shown in Fig. 5, transcription of these genes was abolished in ∆*ompR* and ∆*ompR*/G63S OmpR strains, indicating that phosphorylated OmpR is indispensable for activating *csgD* and *dtpA* gene expression. *csgD* is a transcriptional regulator that controls curli assembly. Curli are proteinaceous fibers and their production will enhance cell-cell-interaction during biofilm formation and colonization. A mechanistic study had already demonstrated the role of *ompR* in curli biosynthesis^32^.

DtpA belongs to the peptide transportation family, and functions in proton-dependent transport of tripeptides^33^. Bacteria without *dtpA* gene showed a higher survival rate under treatment of antibacterial phosphonpeptide alafosfalin compared with wild type^34^. In order to investigate the efficiency of antibacterial peptide transportation in our transconjugants, the sizes of inhibition zones under various concentrations of alafosfalin were compared (Table 2). No inhibition zone was formed by NDM-TC at a concentration of alafosfalin less than 50 mM, and a very weak inhibitory effect of alafosfalin was observed at 100 mM. The enhanced survival of NDM-TC under the treatment of alafosfalin suggested the failure of *dtpA* activation by G63S OmpR shut down the peptide transportation, leading to antibacterial peptide resistance. Our results indicated that mutation in *ompR* not only repressed porin transcription but also altered other metabolic pathways, which in turn promoted bacterial resistance including to antibiotics and antibacterial peptides.

**Table 2:** Sizes of inhibition zone of transconjugants NDM-T21a and NDM-TC under various concentrations of alafosfalin.

Concentration of alafosfalin (mM)	Diameter of inhibition zone (mm)	
	NDM-T21a	NDM-TC
0	NA	NA
25	9.0 ± 0.3	NA
50	10.0 ± 0.3	NA
100	12.3 ± 0.3	7.0 ± 1.0
200	17.7 ± 0.6	10.3 ± 0.6
500	20.7 ± 1.1	15.3 ± 0.6

Sizes of halo are measured with biological triplicates to determine standard deviations.

NA, no inhibition zone observed.

## Discussion

Continuous antibiotic exposures have been shown to introduce different selective pressures on bacteria which eventually alter drug susceptibilities and even species diversities^35,36^. Previous studies focused on the concentration and duration of antibiotic treatment that induce bacterial resistance^37,38^ as well as the influence of antibiotic exposures to horizontal gene transfer, in particular their effects on bacterial conjugation^15,39^. Nevertheless, the combinatorial effect from both conjugation and antibiotic stimulation to the evolutionary pathway of bacteria carrying drug resistance plasmid remains unclear, provided that multiple events are commonly found in the microbial world. In this study, we set out to investigate the bacterial response under the pressure of both conjugation and antibiotic stimulation. To prevent the influence on conjugation dynamics and efficiency, exposure of various concentrations of antibiotics was conducted in the selection process rather than the mating process. Moreover, the maximum antibiotic selection dosage was similar to the highest concentration (2.8 µg/mL) in patients’ pleural tissues after meropenem injection^40^, in order to mimic the clinical setting at the hospital. According to literature, there is about 0.4 µg/g of meropenem remained in the colon after 6 hours of infusion^41^. For urinary tract, there will be more than 1 µg/mL of meropenem in the urine after 12 hours^42^. In our work, we found that the bacterial survival was drastically reduced under selection of higher meropenem concentration. Nevertheless, these survival clones consequently evolved into bacteria with resistance to various kinds of carbapenems. This result demonstrated that emerging antibiotic resistance is very complex and could be achieved under influence of different bacterial events such as plasmid conjugation and antibiotic selection pressure. Our study suggests that antibiotic treatment immediately after conjugation determines the evolutionary pathway of bacteria.

In our transcriptomes analysis, one of the resistance mechanisms was attributed to a mutation on *ompR*, which represses the transcription of porins as well as other metabolic pathways. There were a few clinical reports of genetic variations on *ompR*^9,43^. This may due to additional fitness cost imposed by mutant OmpR. It was shown that deletion of *ompR* caused a 20% reduction in growth rate, while the *ompCF* double mutant only led to a 10% decline^27^. We also used the growth rates to compare their fitness (Fig. S7) and the doubling time of *E. coli* J53, NDM-T21a and NDM-TC were 33, 37 and 45 min respectively. Such reduction in fitness possibly lower bacterial survival, resulting in less frequent isolation of *ompR* mutants. Up till now, a few point mutations in *ompR* were found under long-term antibiotic selection. In particular, a G63V mutant OmpR was recently identified from an *E. coli* ST131 clinical isolate^27,29,43^, suggesting glycine 63 may be a mutation hot spot in OmpR to modulate porin expression.

OmpR consists of the N-terminal regulatory and C-terminal DNA-binding domains. Genetic variation restricts the phosphorylation of the N-terminal domain of OmpR, thereby affecting the capability for DNA binding and leading to porin deficiency. A novel mutation G63S, which locates to the N-terminus of OmpR and is in close proximity to the phosphorylation site Asp-55 of OmpR (Fig. 4) was identified, and its regulation to carbapenem resistance was characterized in this study. Glycine-to-serine substitution introduces one more hydroxymethyl group in the side chain of the amino acid, and this extra hydroxyl group possibly restricts the approach of EnvZ to the phosphorylation site of OmpR due to charge and steric hindrance effects. G63S OmpR fail to activate the transcription of *ompC* and *ompF* as they could not bind to their promoter region. Thus, the antibiotic influx will be reduced and the bacteria become drug resistant.

We have identified several OmpR-regulons including *csgD* and *dtpA* via RNA-Seq. DtpA is a member of the tripeptide transportation system that controls the uptake of small peptides and peptidomimetics. We demonstrated that *dtpA* was regulated by OmpR, and a mutation in *ompR* reduced the influx of the antibacterial dipeptide, alafosfalin. Alafosfalin can selectively inhibit peptidoglycan biosynthesis^44^ and has been proposed for treating urinary tract infection^45^. This peptide displays promising antimicrobial properties and has been successfully utilized to isolate *Salmonella* from clinical samples^46^. Although alafosfalin is not currently prescribed for medical use, antimicrobial peptides have become a popular alternative to combat drug-resistant infections due to their broad anti-inflammatory activities and high efficiency^47^. Since peptide transportation is controlled by *ompR*, the function of peptide-based treatment may be attenuated in pathogens with mutations in *ompR*. The effect of peptide-based medicines in *ompR* or *dtpA* mutants remains elusive.

The role of OmpR in bacterial pathogenicity is still controversial. OmpR in *Salmonella enterica* serovar Typhimurium has been found to activate *ssrA*-*ssrB*, a regulatory system in Pathogenicity Island 2 that is responsible for replication inside macrophages and systematic infection in mice. *Salmonella* without *ompR* therefore fail to survive inside macrophages^48^. Moreover, deletion of *ompR* in uropathogenic *E. coli* (UPEC) lead to a significant reduction of bacterial survival in murine bladders and kidneys^49^, which indicated the significance of OmpR in urinary tract infection. However, the loss of porins may also be beneficial for bacterial infection in some cases. In *K. pneumoniae*, loss of porins OmpK 35 and OmpK 36, homologs of OmpC and OmpF, respectively, showed different protein composition of outer membrane vesicles. These vesicles were found to induce secretion of extremely low levels of proinflammatory cytokine^50^. Indeed, low cytokine secretion may be an evasion mechanism for bacteria to escape from the host immune response. Since *ompR* is responsible for activating transcription of *ompC* and *ompF*, the *ompR* mutant may also produce vesicles with less proinflammatory cytokines in *K. pneumoniae*. The influence of *ompR* mutations on the protein profile of vesicles as well as their role in bacterial pathogenicity in different pathogens should be explored in the future.

In summary, our findings have revealed the potential risk of bacterial conjugation combined with antibiotic exposure over a short period of time. The reduced efficiency of plasmid transfer together with the elevated MIC in survival bacterial clones dictated a more complicated mechanism of evolving antibiotic resistance in the bacteria. The identification of OmpR mutants through transcriptome analysis and their regulatory effect with respect to reduced porin expressions indicated one of the mechanistic pathways leading to carbapenem resistance of bacteria. Further understanding of OmpR-regulons obtained from RNA-Seq provided more information on the functional roles of *ompR* in bacterial metabolism and virulence. Our results demonstrated the influence of gene moderation by a master regulator, *ompR*, in various metabolic pathways, which in turn promote antimicrobial resistance.

## Electronic supplementary material

Supplementary Information

Dataset S1.

Dataset S2.

Dataset S3.
