# Supplementary material for: Fine-tuning carbapenem resistance by reducing porin permeability of bacteria activated in the selection process of conjugation
Source: Sci Rep. 2018 Oct 15;8:15248. doi: 10.1038/s41598-018-33568-8 (PMC6189183; doi:10.1038/s41598-018-33568-8)
Supplement: Supplementary file 1 — Supplementary Information [file 41598_2018_33568_MOESM1_ESM.pdf]

Fine-tuning carbapenem resistance by reducing porin permeability of bacteria activated in the selection process of conjugation

Hoi-Kuan Kong[1], Qing Pan[1], Wai-U Lo[2], Xuan Liu[1], Carmen OK Law[1],

Ting-fung Chan[3], Pak-Leung Ho[2], Terrence Chi-Kong Lau[1,#]

[1] Department of Biomedical Sciences, City University of Hong Kong, Hong Kong Special Administrative Region, People's Republic of China.

[2] Department of Microbiology and Carol Yu Centre for Infection, The University of Hong Kong, Hong Kong Special Administrative Region, People's Republic of China.

[3] School of Life Sciences, The Chinese University of Hong Kong, Hong Kong Special Administrative Region, People's Republic of China.

[#] Address correspondence to Terrence Chi-Kong Lau, chiklau@cityu.edu.hk.

Supplementary information

TABLE S1. *E. coli* strains and plasmids used in this study.

Strain	Genotype	Ref or source
BW25113	*lacI*$^{+}$*rrnB*$_{T14}$ Δ*lacZ*$_{WJ16}$ *hsdR514* Δ*araBAD*$_{AH33}$ Δ*rhaBAD*$_{LD78}$ rph-1 Δ(*araB–D*)*567* Δ(*rhaD–B*)*568* Δ*lacZ4787*(::*rrnB-3*) *hsdR514 rph-1*	(1)
DH5α	*F− φ80dlacZΔM15 Δ(lacZYA-argF)U169 deoR recA1 endA1 hsdR17*(*rk− mk+*) *phoA supE44 λ−* thi-1 gyrA96 relA1	Lab stock
BL21DE3	*fhuA2 [lon] ompT gal (λ DE3) [dcm] ∆hsdS λ DE3 = λ sBamHIo ∆EcoRI-B int::(lacI::PlacUV5::T7 gene1) i21 ∆nin5*	Lab stock
MG1655	*F*$^{-}$*, λ*$^{-}$*, rph-1*	Lab stock
J53	F^{+} *met pro Azi*^{r}	Lab stock
JW3368	BW25113 *ompR*::*kan*, Kn^{r}	(1)
JW2203	BW25113 *ompC*::*kan*, Kn^{r}	(1)
JW0912	BW25113 *ompF*::*kan*, Kn^{r}	(1)
Plasmid		
pET28a	phage T7 promoter, Kn^{r}	Lab stock
pGEX-6P-1	Tac promoter, Ap^{r}	Lab stock
pNN387	Low copy vector, Cm^{r}	Gift from Kunihiko Nishino
pTL39	*ompR* +1 to +720 in pET28a *BamH* I/*Hind* III*,* Kn^{r}	This study
pTL40	pTL39 with G63S, Kn^{r}	This study
pTL63	pTL39 with D55A, Kn^{r}	This study
pTL65	*envZ* +540 to +1353 in pGEX-6P-1 *BamH* I/*Xho* I, Ap^{r}	This study
pTL74	*ompR* -250 to + 840 in pNN387 *Hind* III/*Not* I, Cm^{r}	This study
pTL75	pTL74 with G63S, Cm^{r}	This study

Reference

1. Baba T, Ara T, Hasegawa M, Takai Y, Okumura Y, Baba M, Datsenko KA, Tomita M, Wanner BL, Mori H. 2006. Construction of Escherichia coli K-12 in-frame, single-gene knockout mutants: the Keio collection. Mol Syst Biol 2:2006 0008.

TABLE S2. Primers used in this study.

Primer name	Sequence 5'→3'
gapA-F	GCTCGTAAACACATCACCGC
gapA-R	CGATGTCCTGGCCAGCATAT
ompC-F	GACCTACATGCGTCTTGGCT
ompC-R	GCTGTTGCCCTGGATCTGAT
ompF-F	GACCGTACCAACCTGCAAGA
ompF-R	GTTCGCTGCCAGGTAGATGT
ompR-F	GCCTGCTGACTCGTGAATCT
ompR-R	CGGGTTGCTCTGACTACGAA
dtpA-F	CGTTGTAGGCCTGCTGATCA
dtpA-R	GTCAGCAGCAGGTTACGGTA
csgD-F	CAATGGGTTGCAAGGCGTC
csgD-R	GGGCTGATTCCGTGCTGTTA

TABLE S3. Meropenem MIC of porin mutants carrying pNDM-HK.

Strain	Genotype	MIC (μg/mL)<br>Meropenem
BW25113	Wild-type	0.032
JW3368	△*ompR*	0.032
JW2203	△*ompC*	0.023
JW0912	△*ompF*	0.047
BW25113/pNDM-HK	Wild-type	6
JW3368/pNDM-HK	△*ompR*	32
JW2203/pNDM-HK	△*ompC*	4
JW0912/pNDM-HK	△*ompF*	6
JW3368/pNDM-HK complemented with WT OmpR	/	12
JW3368/pNDM-HK complemented with G63S OmpR	/	24

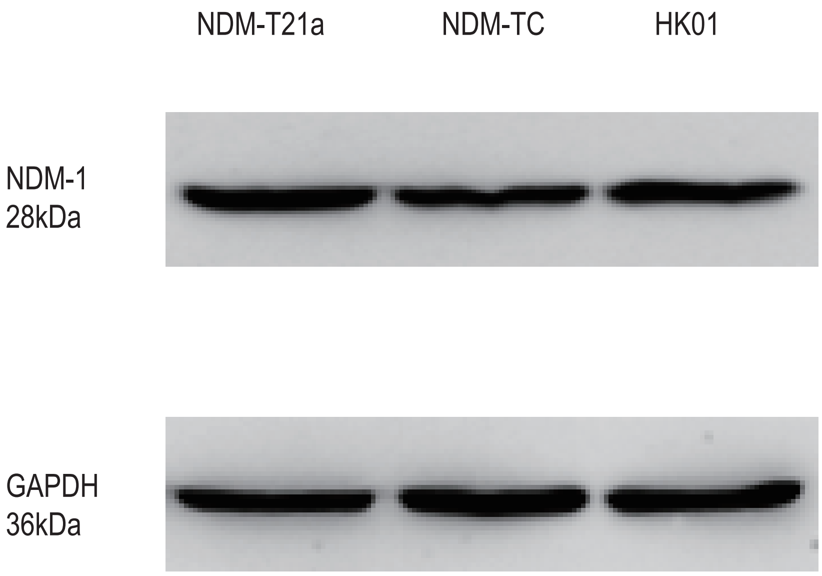

FIG S1. NDM-1 and GAPDH protein level in donor strain HK01, transconjugants NDM-T21a and NDM-TC by Western blot. GAPDH was probed as the loading control.

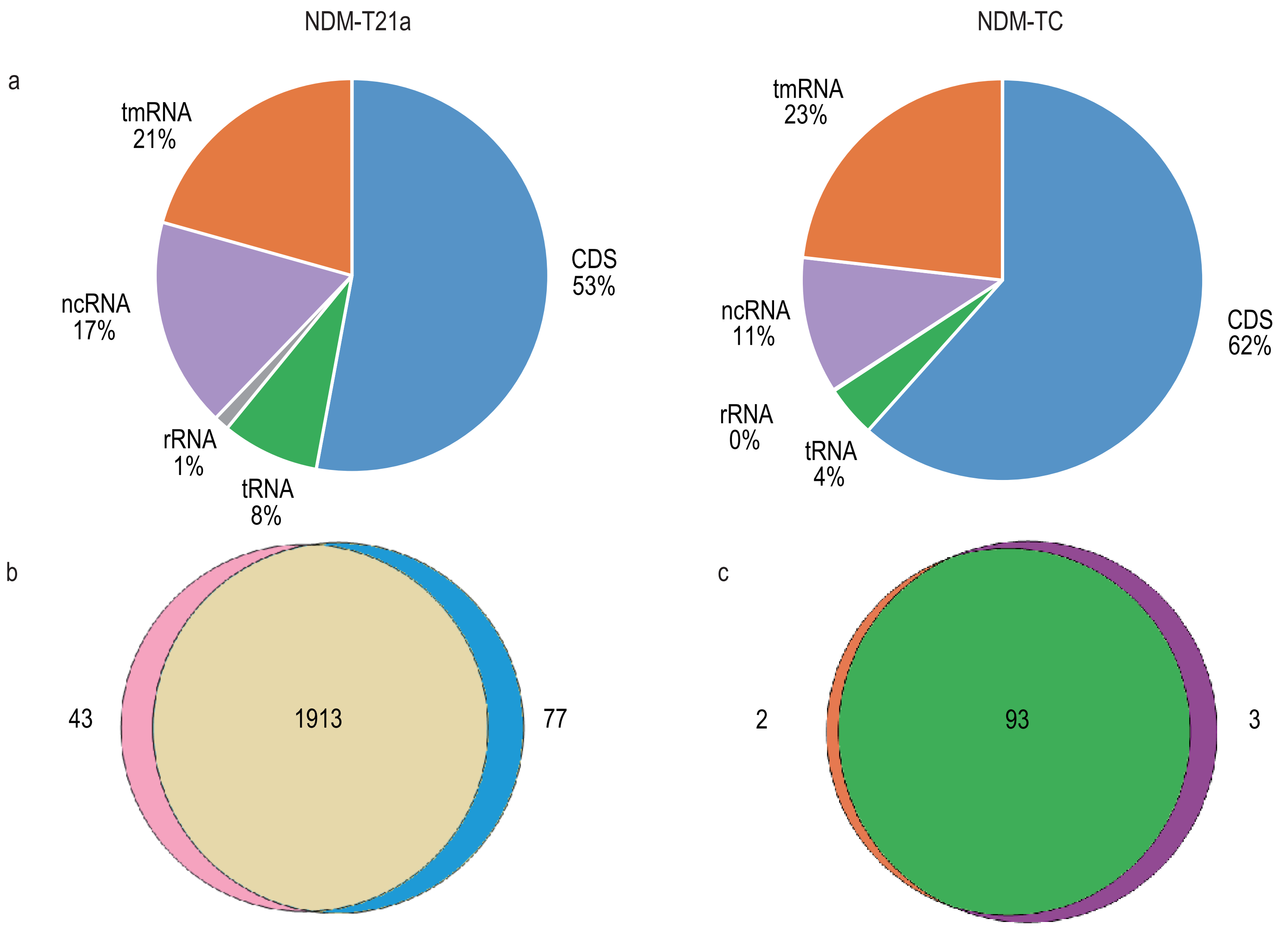

FIG S2. Sequencing statistics of NDM-T21a and NDM-TC. (a) Reads mapping to coding region (blue), rRNA (grey), tRNA (green), tmRNA (orange) and non-coding RNA (purple). (b) Number of chromosome-encoded genes of NDM-TC that are upregulated (blue), downregulated (pink) and unchanged (grey) compared with NDM-T21a. (c) Number of plasmid-encoded genes of NDM-TC that are upregulated (purple), downregulated (orange) and unchanged (green) compared with NDM-T21a.

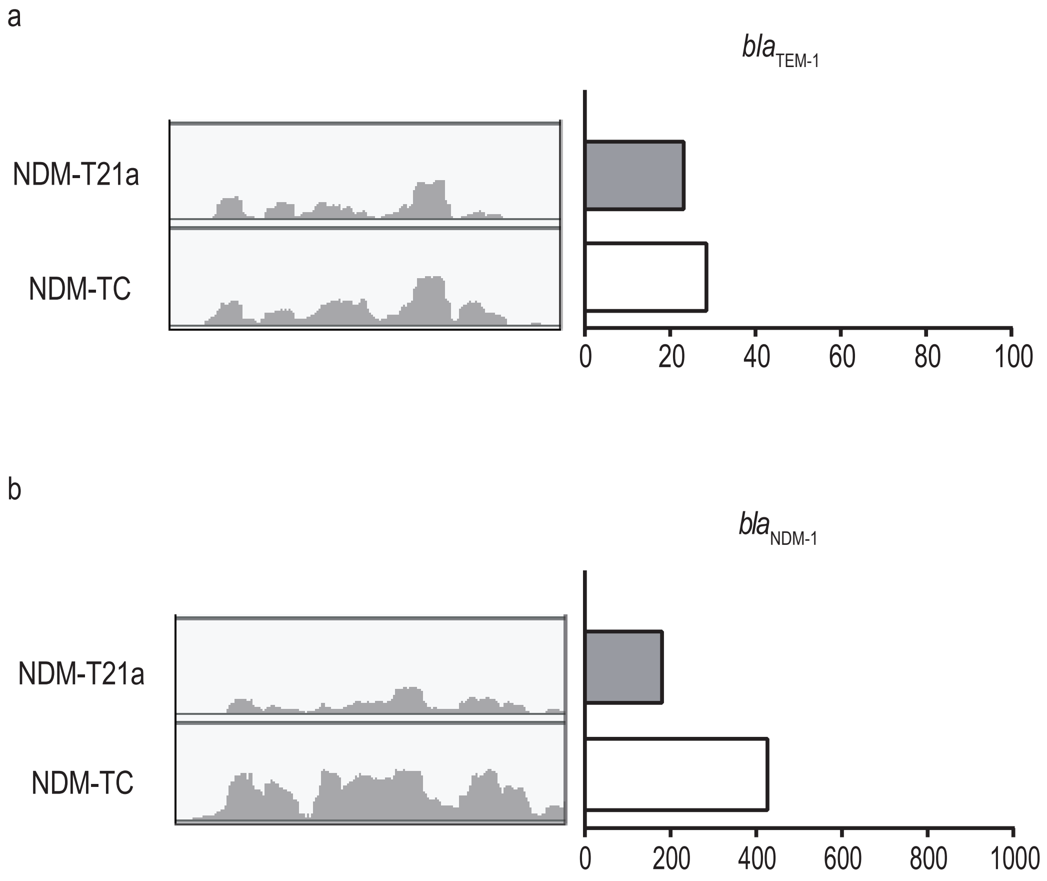

FIG S3. Read-count of *bla*$_{TEM-1}$ and *bla*$_{NDM-1}$. Number of read count of genes (a) *bla*$_{TEM-1}$ and (b) *bla*$_{NDM-1}$ obtained in RNA-Seq of NDM-T21a (grey column) and NDM-TC (white column) is posted. IGV-captured diagrams with the same scale are placed next to the bar chart.

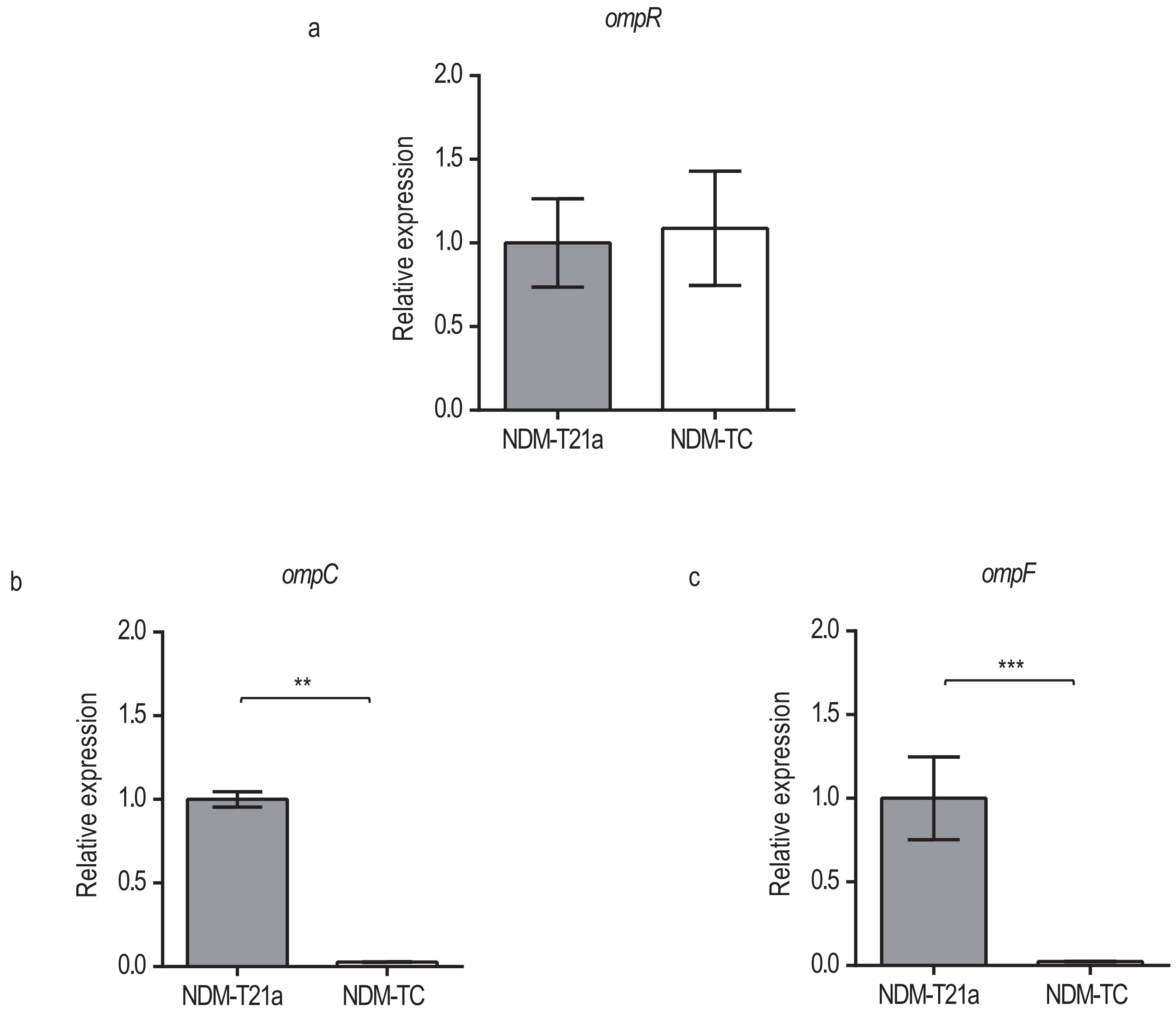

FIG S4. Transcription level of porin genes obtained by qRT-PCR. Expression levels of (a) *ompR*, (b) *ompC*, and (c) *ompF* in transconjugants NDM-T21a (grey column) and NDM-TC (white column) obtained by qRT-PCR. Vertical error bars show the standard deviations of biological triplicates. The expression level obtained in NDM-T21a is set as reference for comparison. *P-value<0.05; **P-value<0.01; ***P-value<0.001.

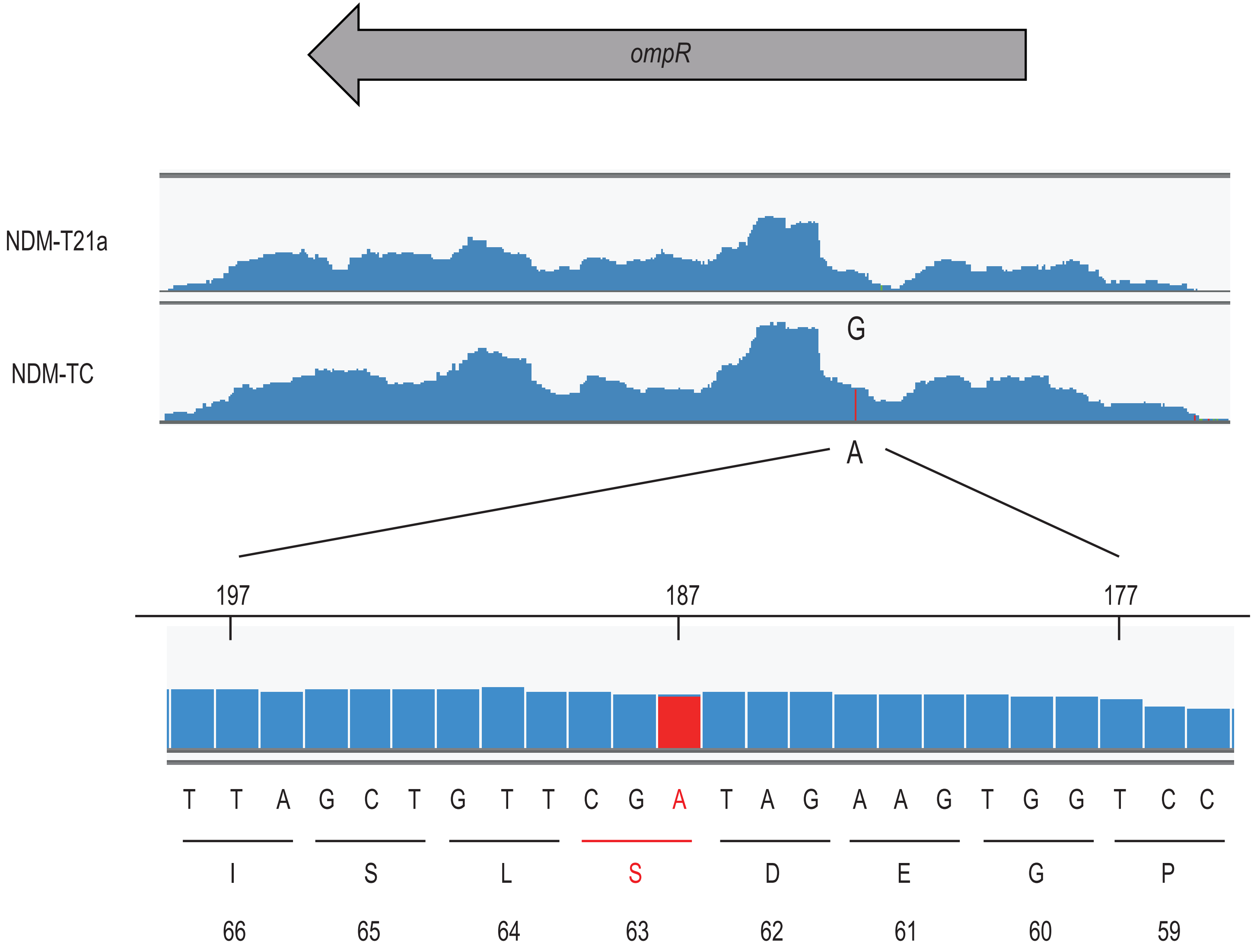

FIG S5. IGV diagram of *ompR* gene in transconjugants NDM-T21a and NDM-TC. Region flanking nucleotides 177-197 of *ompR* in NDM-TC are zoomed in and the corresponding amino acids are placed below the DNA sequences.

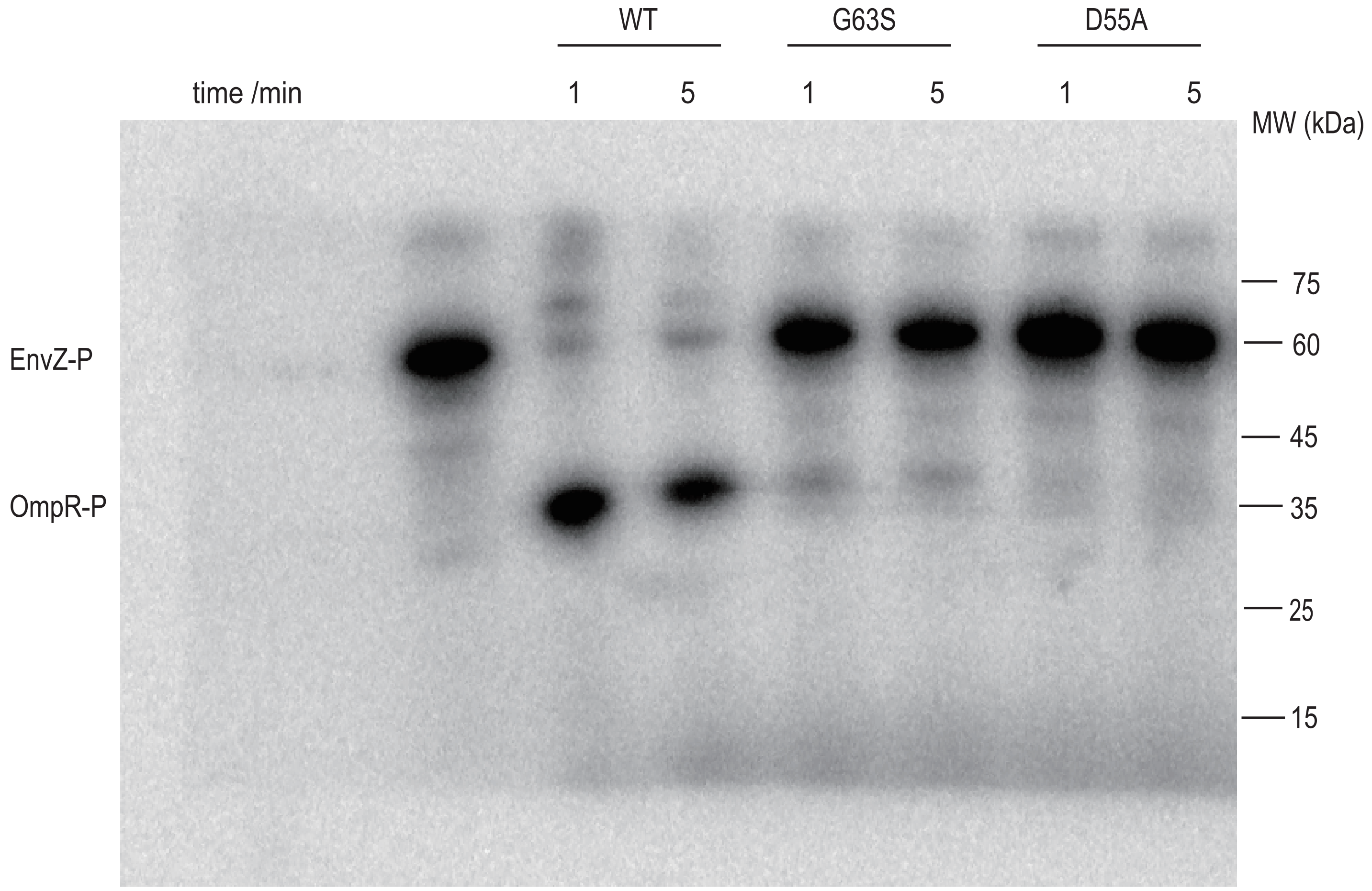

FIG S6. Full image of electrophoretic gel Fig 3.

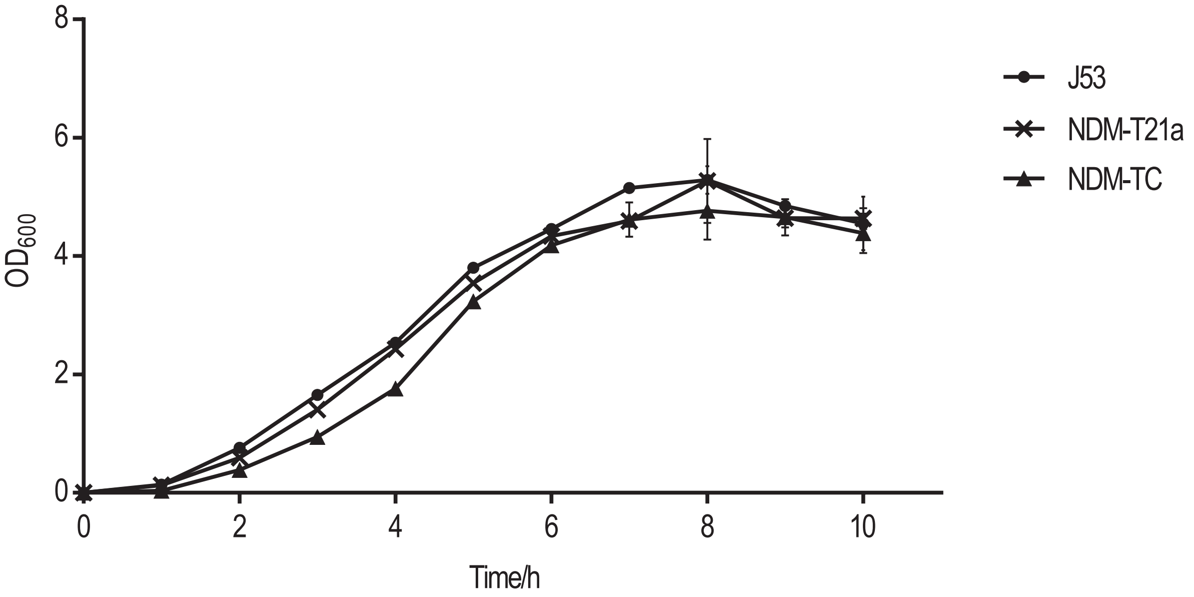

FIG S7. Growth curve of *E. coli* J53, NDM-T21a and NDM-TC. Vertical error bars represented standard deviation of biological triplicate.
